# Occult neck metastasis in salvage laryngectomy: a road map for super-selective neck dissection

**DOI:** 10.1017/S0022215124000720

**Published:** 2024-09

**Authors:** Ahmed Youssef, Austin Milton, Rusha Patel, Rachad Mhawej, Nilesh Vasan, Greg Krempl

**Affiliations:** 1 Department of Otolaryngology & Head and Neck Surgery, University of Oklahoma Health Sciences Center, Oklahoma City, USA; 2 Department of Otolaryngology & Head and Neck Surgery, Alexandria University, Alexandria, Egypt

**Keywords:** neoplasms, health planning guidelines

## Abstract

**Objective:**

This study aimed to solve the debate over the role and extent of neck dissection to treat any occult nodal metastasis in patients undergoing salvage laryngectomy for recurrent and/or residual squamous cell carcinoma of larynx.

**Methods:**

This was a retrospective study over a time frame of 6 years (2016–2022) of 74 patients who underwent bilateral neck dissection and salvage laryngectomy for recurrent or persistent disease with N0 neck. We calculated the incidence of occult nodal metastasis in ipsilateral and contralateral neck.

**Results:**

Incidence of ipsilateral neck disease was calculated as 8.11 per cent and it was 0 per cent in contralateral neck. Regarding ipsilateral nodal level distribution, level II was the highest at 6.76 per cent, followed by level III at 5.41 per cent. There was 0 per cent metastasis in levels IV and IIb.

**Conclusion:**

In patients undergoing salvage laryngectomy with N0 neck, ipsilateral super selective neck dissection is considered a convenient and oncologically safe option to treat the neck.

## Introduction

The Veterans Affairs and Radiation Therapy Oncology Group 91-11 trials established organ preservation with chemo-radiotherapy as the standard of care for locally advanced laryngeal cancers, with surgical salvage reserved for those with recurrent or residual disease.^1–3^ In cases of locally recurrent or residual squamous cell carcinoma (SCC) of the larynx, there is debate over the role of elective neck dissection for clinically and radiologically negative nodal metastasis, defined as N0 neck. Dissection in the irradiated neck is associated with increased post-operative complications such as wound dehiscence and possible vessel blowout.

Previous studies have had mixed results for the incidence of occult neck metastasis (ranging from 0 to 28 per cent) with salvage laryngectomy.^4–9^ A 2019 meta-analysis by Lin *et al*. reviewed 922 patients who underwent elective neck dissection and 276 who were observed.^10^ The study revealed an overall occult nodal metastasis rate of 14 per cent, which falls below the historically quoted rate of more than 20 per cent reported by Weiss *et al*.^10,11^

Regarding survival, Hilly *et al*. found a significant difference in disease-free survival and overall survival of patients with primary T3 and/or T4 tumours when comparing neck dissection to observation. Disease-free survival at 5 years was more than 60 per cent for the elective neck dissection group and less than 20 per cent for the observation group. Similar findings were found in overall survival. However, there was no benefit seen for elective neck dissection compared with observation in the setting of salvage laryngectomy, although there was a prognostic value.^12^

On the other hand, Freiser *et al*. found that disease-free survival at 2 years was doubled (60 per cent) for pathological N0 status compared with pathological N+ status (30 per cent). Overall survival at 2 years was 75 per cent with pathological N0 status and 50 per cent with pathological N+ status.^13^ Differences in survival outcomes in salvage laryngectomy with elective neck dissection may depend on radiation and surgical technique, and the survival benefit remains to be clarified.

Numerous studies have been performed in the last 20 years to determine whether elective neck dissection is required with salvage laryngectomy, but none have given clear recommendations to address two important questions: whether contralateral neck dissection is necessary and whether there is value in a super-selective neck dissection sparing levels IIB and IV in these patients.

Our study assessed the incidence of occult lymph nodes metastasis in salvage laryngectomy for patients who underwent bilateral neck dissection. Our objectives were (1) to determine the rate of contralateral nodal disease in elective neck dissection for salvage laryngectomy and (2) to determine nodal levels of involvement during elective neck dissection for salvage laryngectomy.

## Materials and methods

A retrospective review was conducted of patients undergoing bilateral neck dissection during salvage laryngectomy for recurrent or persistent disease without clinical or radiological evidence of positive cervical lymph nodes (N0) over a period of 5 calendar years starting from 2017.

Demographic data were collected, including primary tumour site and stage, prior treatment (radiation or chemoradiation), and presence and location of nodal deposits. Neck dissections were evaluated by a pathologist using serial sectioning technique. Data were presented as percentages of the whole.

## Result and analysis

Seventy-four patients were identified during the study period. All patients received chemo-radiotherapy as 70 Gy over 35 fractions over period of 7 weeks with concomitant cisplatin on days 1, 21 and 42. Pretreatment stage was T3 N0 M0 with different subsites involved (5 cases were supraglottic, 67 were trans-glottic and the remaining 2 were subglottic). Radiation dose was given as 50 Gy for both sides of the neck in 5 patients while the same dose was offered on the ipsilateral side in all remaining patients.

All patients underwent post-treatment positron emission tomography-computed tomography scanning 12 weeks after completion of treatment that showed residual disease in 49 patients. Recurrence was reported in the remaining 25 patients after 12 months of completion of treatment. Late recurrence was reported in 9 patients as late as 30 months after end of their treatment.

All residual tumours were staged as T3 while nine of the recurrent cases were T4.

In total, 62 patients underwent bilateral neck dissection for levels II–IV. The 12 remaining patients with no supraglottic major involvement underwent ipsilateral neck dissection as surgeon preference to reduce operative time during the coronavirus disease 2019 pandemic. Only 27 patients underwent level VI (paratracheal) lymph node dissection. Paratracheal lymph node dissection was performed in all 25 recurrent patients and 2 patients with subglottic residual tumour.

Eight of the 74 cases had positive ipsilateral nodal disease, with an incidence rate of 10.8 per cent. Ipsilateral neck disease was limited to levels IIa and III (Table 1). Four patients had ipsilateral neck disease involving level II, while three patients had isolated neck metastasis involving only level III. One patient had both levels II and III involved on the same side of tumour. There was zero incidence of contralateral occult neck metastasis.

**Table 1.** Incidence of occult metastasis

Level	Number of cases (*n*)	Number with occult metastases	Incidence of occult metastasis (%)
IIA S	74	5	6.8
IIB S	74	0	0
III S	74	4	5.4
IV S	74	0	0
II C	62	0	0
III C	62	0	0
IV C	62	0	0
VI	27	1	3.6

S = ipsilateral; C = contralateral

Paratracheal lymph nodes were only positive in one patient with a previous history of hemi-laryngectomy followed by chemo-radiotherapy for positive margins who presented later with local laryngeal recurrence with hypopharyngeal involvement. All patients were followed at a head and neck cancer surveillance clinic following the five-year rule. There was no stomal or tumour recurrence on either ipsilateral or contralateral necks. Earlier 2017/2018 patients had been discharged from service as they were cancer-free for five years.

## Discussion

In the era of laryngeal conservation, there has been uncertainty about the benefit of elective neck dissection in N0 patients undergoing salvage laryngectomy. Data comparing rates of occult nodal disease, specifically contralateral disease, are variable. Some studies report no occult nodal metastasis in the contralateral neck while other studies did not comment on differences in laterality.^13–16^ The meta-analysis by Lin *et al*. recommended further study to compare unilateral and bilateral elective neck dissection.^10^

In a study by Basheeth *et al*., 20 patients undergoing salvage laryngectomies underwent unilateral elective neck dissection and 18 underwent bilateral elective neck dissection. Only 3 patients (8 per cent) had nodal positivity and there was no difference in regional control for unilateral versus bilateral dissection, but the complication rate was over twice as high for bilateral node dissection (67 per cent) compared with unilateral node dissection (30 per cent).^6^

In our data, there was zero percentage of contralateral nodal involvement in all patients who underwent salvage laryngectomy. Interestingly, there was no nodal involvement in ipsilateral levels IIb and 4. These findings raised the question of the utility of complete ipsilateral neck dissection versus selective neck dissection in these patients.

Regarding the primary anatomical subsite involvement, a review of prior literature revealed a higher rate of occult nodal metastasis in patients with recurrences that were supraglottic (24 per cent) and transglottic (17 per cent) as well as recurrent T3 and T4 tumours.^10,16^ Both primary and recurrent supraglottic SCC consistently have a higher rate of lateral neck metastasis than other laryngeal subsites.^17,18^ Several authors have recommended routine elective neck dissection for supraglottic recurrence and for T3 and/or T4 tumours based on higher rates of occult metastasis compared with glottic recurrence.^8,15^ However, none of these studies have directly addressed whether bilateral dissection is needed.

Birkeland *et al*. studied 203 patients treated with elective neck dissection and found a significantly higher rate of occult metastasis in supraglottic recurrence compared with glottic recurrence (28 *vs* 10 per cent, respectively). They also found that T4 status was significantly associated with an increased rate of occult metastasis in both univariate (34 per cent rate of occult nodal metastasis) and bivariate analyses when paired with the supraglottic subsite (50 per cent rate of occult nodal metastasis). The study recommended consideration of bilateral level II and III dissection, while also acknowledging that positive nodes from supraglottic primaries were most frequently at ipsilateral levels II and III (17 and 16 per cent) and positive nodes from glottic primaries were most frequently located at ipsilateral and contralateral paratracheal nodes (11 and 9 per cent) which may or may not be dissected during the laryngectomy.^16^

- There is a debate about the role and extent of neck dissection in patients undergoing salvage laryngectomy with N0 neck
- Bilateral neck dissection could be avoided in salvage laryngectomy with pre-operative N0 neck proved clinically and radiologically using positron emission tomography-computed tomography scan
- Ipsilateral super-selective neck dissection is considered a safe option that avoids unnecessary dissection of levels IIb or IV and could avoid patient potential morbidities and reduce the time of surgery
- Paratracheal lymph node dissection is advised in subglottic or advanced T4 recurrence of squamous cell carcinoma of the larynx

Koss *et al*. also found a significantly higher rate of occult nodal metastasis in supraglottic (60 *vs* 9 per cent in glottic) and transglottic recurrences (30 per cent), but the study did not evaluate laterality.^15^

In keeping with the above findings, we found occult nodal metastasis in all supraglottic recurrence with either epiglottis or aryepiglottic fold as primary. All of cases with positive nodal disease had advanced T3 and/or T4 recurrences.

Pennings *et al*. treated 93 clinically N0 salvage laryngectomy patients with super-selective elective neck dissection (levels II and III only) and there was a 0 per cent recurrence rate in cases that were pathologically N0. The authors recommended level II and III super-selective elective neck dissection as a staging procedure that allows the possibility of sending suspicious nodes for intra-operative frozen section.^8^ In agreement with their findings, our results showed occult neck disease was found in both level II and III on the same side of the primary site of recurrence.

In contrast to the above studies, the Brazilian Head and Neck Cancer Study Group found 5 cases of level IV metastasis without level II or III involvement among 111 patients who underwent primary or salvage laryngectomy. However, the rate of occult neck metastasis was only 6 per cent among salvage laryngectomy patients who had received radiotherapy compared with 24 per cent for primary laryngectomy.^17^

Surgeons opting to forgo contra-lateral lymph node dissection should be aware that paratracheal lymph nodes can be positive without lateral neck node positivity, but there is a clear association between the two. In a study of 210 clinically N0 patients receiving salvage laryngectomy, paratracheal lymph nodes metastasis was associated with lateral neck node involvement (*p* = 0.001), but a significant portion (6 out of 11) had positive paratracheal nodes and negative lateral neck nodes. Based on these findings, it appeared that initial radiotherapy created an unusual route of lymphatic spread.^18^

In addition, a study by Weber *et al*. of 141 patients undergoing primary laryngectomy recommended elective paratracheal lymph nodes dissection based on a 17 per cent incidence of positive nodes (27 per cent with subglottic extension). There was also prognostic value, with an 87 per cent overall survival rate without paratracheal node metastasis whereas none of the patients with paratracheal metastasis survived beyond 42 months.^19^

A 2022 meta-analysis of 838 patients found a 20.7 per cent rate of paratracheal lymph node positivity in primary total laryngectomy and 8.7 per cent positivity in salvage total laryngectomy, recommending further large studies to establish evidence-based recommendations.^20^ There is also a clear association between paratracheal lymph nodes and stomal recurrence, especially in cases of extra-nodal extension, which paratracheal nodes seem prone to, potentially due to their small size and thin capsule.

Consequently, paratracheal lymph node dissection with primary or salvage laryngectomy is routinely performed by some surgeons. However, in our series, paratracheal lymph node dissection was performed in 27 cases with only 1 case positive. That patient had a positive history of previous hemi-laryngectomy with post-operative chemo-radiotherapy for positive margins, then was presented with recurrence. It is possible that prior intervention with positive margins might create that unusual route of spread to the paratracheal lymph nodes. Paratracheal lymph node involvement in salvage laryngectomy remains to be studied.

## Conclusion

This study showed that routine bilateral neck dissection in salvage laryngectomy patients with an N0 neck may be able to be avoided. In addition, the data suggested that super-selective node dissection for ipsilateral levels IIa and III may be an option to be explored in this patient population. Paratracheal lymph node dissection is recommended in recurrences with previous surgical intervention or in subglottic region involvement. We recommend further multi-institutional collaboration regarding the analysis of their findings to both solidify our findings and to provide future treatment recommendations in this population.
